# Supplementary material for: Quantifying the topographical structure of rocky and coral seabeds
Source: PLoS One. 2024 Jun 6;19(6):e0303422. doi: 10.1371/journal.pone.0303422 (PMC11156299; doi:10.1371/journal.pone.0303422)
Supplement: S1 Appendix — (PDF) [file pone.0303422.s001.pdf]

S1 Appendix. Matlab code.

```
function TOPOGRAPHY_METRICS

cd /home/damien/DATA/RECHERCHE/COCODYROCK/PROFILE_PLOS/

%%%%%%%%%%%%%%%%%%%%%%%%%%%%%%%%%%
% Processing parameters
%%%%%%%%%%%%%%%%%%%%%%%%%%%%%%%%%%

dx=0.05;
Lbathy=20;

%%%%%%%%%%%%%%%%%%%%%%%%%%%%%%%%%%
% SOKOA
%%%%%%%%%%%%%%%%%%%%%%%%%%%%%%%%%%


P1=load('SokCS1.dat');
P2=load('SokCS2.dat');
P3=load('SokCS3.dat');
P4=load('SokCS4.dat');
P5=load('SokCS5.dat');
P6=load('SokCS6.dat');

[AV_Sok,STD_Sok,SK_Sok,KURT_Sok,ENT_Sok,ES_Sok,LR_Sok,SF_Sok
   ,COV_Sok]=Metrics(dx,Lbathy,P1,P2,P3,P4,P5,P6)

tmp=load('SokAL1.dat');
Xal1=tmp(:,1);
Zal1=tmp(:,2);
tmp=load('SokAL2.dat');
Xal2=tmp(:,1);
Zal2=tmp(:,2);
tmp=load('SokAL3.dat');
Xal3=tmp(:,1);
Zal3=tmp(:,2);
tmp=load('SokAL4.dat');
Xal4=tmp(:,1);
Zal4=tmp(:,2);
tmp=load('SokAL5.dat');
Xal5=tmp(:,1);
Zal5=tmp(:,2);
tmp=load('SokAL6.dat');
Xal6=tmp(:,1);
Zal6=tmp(:,2);

% Directionality

Pa1=load('SokAL1.dat');
Pa2=load('SokAL2.dat');
Pa3=load('SokAL3.dat');
Pa4=load('SokAL4.dat');
Pa5=load('SokAL5.dat');
Pa6=load('SokAL6.dat');
```

```
[~,STD_Sok_al,~,~,~,~,~,~,~]=Metrics(dx,Lbathy,Pa1,Pa2,Pa3,
    Pa4,Pa5,Pa6);

DIR_Sok=(mean(STD_Sok)-mean(STD_Sok_al))/(mean(STD_Sok)+mean
    (STD_Sok_al))


%%%%%%%%%%%%%%%%%%%%%%%%%%%%%%%%%%
% ARS EN RE
%%%%%%%%%%%%%%%%%%%%%%%%%%%%%%%%%%

P1=load('Re1.dat');
P2=load('Re2.dat');
P3=load('Re3.dat');
P4=load('Re4.dat');
P5=load('Re5.dat');
P6=load('Re6.dat');

[AV_Re,STD_Re,SK_Re,KURT_Re,ENT_Re,ES_Re,LR_Re,SF_Re,COV_Re
    ]=Metrics(dx,Lbathy,P1,P2,P3,P4,P5,P6)

DIR_Re=NaN;

%%%%%%%%%%%%%%%%%%%%%%%%%%%%%%%%%%
% MAUPITI A
%%%%%%%%%%%%%%%%%%%%%%%%%%%%%%%%%%

P1=load('MaupA1.dat');
P2=load('MaupA2.dat');
P3=load('MaupA3.dat');
P4=load('MaupA4.dat');

[AV_MaupA,STD_MaupA,SK_MaupA,KURT_MaupA,ENT_MaupA,ES_MaupA,
    LR_MaupA,SF_MaupA,COV_MaupA]=Metrics(dx,Lbathy,P1,P2,P3,
    P4)

DIR_MaupA=NaN;

%%%%%%%%%%%%%%%%%%%%%%%%%%%%%%%%%%
% MAUPITI B
%%%%%%%%%%%%%%%%%%%%%%%%%%%%%%%%%%

P1=load('MaupB1.dat');
P2=load('MaupB2.dat');
P3=load('MaupB3.dat');
P4=load('MaupB4.dat');

[AV_MaupB,STD_MaupB,SK_MaupB,KURT_MaupB,ENT_MaupB,ES_MaupB,
    LR_MaupB,SF_MaupB,COV_MaupB]=Metrics(dx,Lbathy,P1,P2,P3,
    P4)


DIR_MaupB=NaN;

%%%%%%%%%%%%%%%%%%%%%%%%%%%%%%%%%%
% MAUPITI C
%%%%%%%%%%%%%%%%%%%%%%%%%%%%%%%%%%
```

```
P1=load('MaupC1.dat');
P2=load('MaupC2.dat');
P3=load('MaupC3.dat');
P4=load('MaupC4.dat');

[AV_MaupC,STD_MaupC,SK_MaupC,KURT_MaupC,ENT_MaupC,ES_MaupC,
   LR_MaupC,SF_MaupC,COV_MaupC]=Metrics(dx,Lbathy,P1,P2,P3,
   P4)


DIR_MaupC=NaN;

%%%%%%%%%%%%%%%%%%%%%%%%%%%%%%%%%%
% NIAU
%%%%%%%%%%%%%%%%%%%%%%%%%%%%%%%%%%

P1=load('Niau1.dat');
P2=load('Niau2.dat');
P3=load('Niau3.dat');
P4=load('Niau4.dat');
P5=load('Niau5.dat');
P6=load('Niau6.dat');

[AV_Niau,STD_Niau,SK_Niau,KURT_Niau,ENT_Niau,ES_Niau,LR_Niau
   ,SF_Niau,COV_Niau]=Metrics(dx,Lbathy,P1,P2,P3,P4,P5,P6)


DIR_Niau=NaN;

%%%%%%%%%%%%%%%%%%%%%%%%%%%%%%%%%%
% BANNEG
%%%%%%%%%%%%%%%%%%%%%%%%%%%%%%%%%%

X=load('BannegX.txt');
Y=load('BannegY.txt');
Z=load('BannegZ.txt');


for iy=1:size(Y,1)

    Zbed=Bathy10th(X(iy,:),Z(iy,:),0.1,Lbathy);
    data=Z(iy,:)-Zbed;
    Z(iy,:)=data;
    indok=find(~isnan(data));
    data=data(indok);
    xdata=X(iy,indok);

if ~isempty(data)
        AV_Ban(iy)=nanmean(data,'all');
        STD_Ban(iy)=std(data,0,'all','omitnan');
        SK_Ban(iy)=skewness(data,1,'all');
        KURT_Ban(iy)=kurtosis(data,1,'all')-3;
```

```
            [p,Edges]=histcounts(data,'Normalization','
               probability');
            ENT_Ban(iy)=-nansum(p.*log2(p));
            ES_Ban(iy)=EffectiveSlope1D(xdata,data);
            LR_Ban(iy)=linear_roughness(xdata,data);
            REC_Ban(iy)=elevational_change(xdata,data);
            SF_Ban(iy)=solid_fraction(xdata,data);
            COV_Ban(iy)=CoefVar(data);
        else
            ES_Ban(iy)=nan;
            LR_Ban(iy)=nan;
            REC_Ban(iy)=nan;
            SF_Ban(iy)=nan;
            AV_Ban(iy)=nan;
            STD_Ban(iy)=nan;
            SK_Ban(iy)=nan;
            KURT_Ban(iy)=nan;
            ENT_Ban(iy)=nan;
        end
        if ES_Ban(iy)>100 | REC_Ban(iy)>100
            ES_Ban(iy)=nan;
            LR_Ban(iy)=nan;
            REC_Ban(iy)=nan;
            SF_Ban(iy)=nan;
            AV_Ban(iy)=nan;
            STD_Ban(iy)=nan;
            SK_Ban(iy)=nan;
            KURT_Ban(iy)=nan;
            ENT_Ban(iy)=nan;
        end

    end

    % Directionality
    for iy=1:size(Y,1)
        STD_x(iy)=std(Z(iy,:),0,'all','omitnan');
    end
    for ix=1:size(Y,2)
        STD_y(ix)=std(Z(:,ix),0,'all','omitnan');
    end

    DIR_Ban=(nanmean(STD_x)-nanmean(STD_y))/(nanmean(STD_x)+
       nanmean(STD_y))



    %%%%%%%%%%%%%%%%%%%%%%%%%%%%%%%%%%
    % PARLEMENTIA A
    %%%%%%%%%%%%%%%%%%%%%%%%%%%%%%%%%%

    P1=load('ParleA_CS_1.dat');
    P2=load('ParleA_CS_2.dat');
```

```
P3=load('ParleA_CS_3.dat');
P4=load('ParleA_CS_4.dat');


[AV_ParleA,STD_ParleA,SK_ParleA,KURT_ParleA,ENT_ParleA,
    ES_ParleA,LR_ParleA,SF_ParleA,COV_ParleA]=Metrics(dx,
    Lbathy,P1,P2,P3,P4)

% Directionality

Pa1=load('ParleA_AL_1.dat');
Pa2=load('ParleA_AL_2.dat');
Pa3=load('ParleA_AL_3.dat');
Pa4=load('ParleA_AL_4.dat');


[~,STD_ParleA_al,~,~,~,~,~,~,~]=Metrics(dx,Lbathy,Pa1,Pa2,
    Pa3,Pa4);

DIR_ParleA=(mean(STD_ParleA)-mean(STD_ParleA_al))/(mean(
    STD_ParleA)+mean(STD_ParleA_al));

%%%%%%%%%%%%%%%%%%%%%%%%%%%%%%%%%%
% PARLEMENTIA B
%%%%%%%%%%%%%%%%%%%%%%%%%%%%%%%%%%

P1=load('ParleB_CS_1.dat');
P2=load('ParleB_CS_2.dat');
P3=load('ParleB_CS_3.dat');

[AV_ParleB,STD_ParleB,SK_ParleB,KURT_ParleB,ENT_ParleB,
    ES_ParleB,LR_ParleB,SF_ParleB,COV_ParleB]=Metrics(dx,
    Lbathy,P1,P2,P3)

% Directionality

Pa1=load('ParleB_AL_1.dat');
Pa2=load('ParleB_AL_2.dat');
Pa3=load('ParleB_AL_3.dat');


[~,STD_ParleB_al,~,~,~,~,~,~,~]=Metrics(dx,Lbathy,Pa1,Pa2,
    Pa3);

DIR_ParleB=(mean(STD_ParleB)-mean(STD_ParleB_al))/(mean(
    STD_ParleB)+mean(STD_ParleB_al));

%%%%%%%%%%%%%%%%%%%%%%%%%%%%%%%%%%%
% RESULTS
%%%%%%%%%%%%%%%%%%%%%%%%%%%%%%%%%%%

% MEAN VALUES
```

```
DataM=[mean(AV_Sok,'omitnan') mean(STD_Sok,'omitnan') mean(
    SK_Sok,'omitnan') mean(KURT_Sok,'omitnan') mean(ENT_Sok,'
    omitnan') mean(ES_Sok,'omitnan') mean(LR_Sok,'omitnan')
    mean(COV_Sok,'omitnan') mean(SF_Sok,'omitnan')  DIR_Sok;
     mean(AV_Re,'omitnan') mean(STD_Re,'omitnan') mean(SK_Re,
        'omitnan') mean(KURT_Re,'omitnan') mean(ENT_Re,'
        omitnan') mean(ES_Re,'omitnan') mean(LR_Re,'omitnan')
         mean(COV_Re,'omitnan') mean(SF_Re,'omitnan')  DIR_Re
        ;
     mean(AV_MaupA,'omitnan') mean(STD_MaupA,'omitnan') mean(
        SK_MaupA,'omitnan') mean(KURT_MaupA,'omitnan') mean(
        ENT_MaupA,'omitnan') mean(ES_MaupA,'omitnan') mean(
        LR_MaupA,'omitnan') mean(COV_MaupA,'omitnan') mean(
        SF_MaupA,'omitnan')  DIR_MaupA;
     mean(AV_MaupB,'omitnan') mean(STD_MaupB,'omitnan') mean(
        SK_MaupB,'omitnan') mean(KURT_MaupB,'omitnan') mean(
        ENT_MaupB,'omitnan') mean(ES_MaupB,'omitnan') mean(
        LR_MaupB,'omitnan') mean(COV_MaupB,'omitnan') mean(
        SF_MaupB,'omitnan')  DIR_MaupB;
     mean(AV_MaupC,'omitnan') mean(STD_MaupC,'omitnan') mean(
        SK_MaupC,'omitnan') mean(KURT_MaupC,'omitnan') mean(
        ENT_MaupC,'omitnan') mean(ES_MaupC,'omitnan') mean(
        LR_MaupC,'omitnan') mean(COV_MaupC,'omitnan') mean(
        SF_MaupC,'omitnan')  DIR_MaupC;
     mean(AV_Niau,'omitnan') mean(STD_Niau,'omitnan') mean(
        SK_Niau,'omitnan') mean(KURT_Niau,'omitnan') mean(
        ENT_Niau,'omitnan') mean(ES_Niau,'omitnan') mean(
        LR_Niau,'omitnan') mean(COV_Niau,'omitnan') mean(
        SF_Niau,'omitnan')  DIR_Niau;
     mean(AV_Ban,'omitnan') mean(STD_Ban,'omitnan') mean(
        SK_Ban,'omitnan') mean(KURT_Ban,'omitnan') mean(
        ENT_Ban,'omitnan') mean(ES_Ban,'omitnan') mean(LR_Ban
        ,'omitnan') mean(COV_Ban,'omitnan') mean(SF_Ban,'
        omitnan')  DIR_Ban;
     mean(AV_ParleA,'omitnan') mean(STD_ParleA,'omitnan')
        mean(SK_ParleA,'omitnan') mean(KURT_ParleA,'omitnan')
         mean(ENT_ParleA,'omitnan') mean(ES_ParleA,'omitnan')
         mean(LR_ParleA,'omitnan') mean(COV_ParleA,'omitnan')
         mean(SF_ParleA,'omitnan')  DIR_ParleA;
     mean(AV_ParleB,'omitnan') mean(STD_ParleB,'omitnan')
        mean(SK_ParleB,'omitnan') mean(KURT_ParleB,'omitnan')
         mean(ENT_ParleB,'omitnan') mean(ES_ParleB,'omitnan')
         mean(LR_ParleB,'omitnan') mean(COV_ParleB,'omitnan')
         mean(SF_ParleB,'omitnan')  DIR_ParleB]

save MeanMetrics.dat DataM -ascii

% STANDARD DEVIATION

DataSTD=[std(AV_Sok,0,'omitnan') std(STD_Sok,0,'omitnan')
    std(SK_Sok,0,'omitnan') std(KURT_Sok,0,'omitnan') std(
```

```
ENT_Sok,0,'omitnan') std(ES_Sok,0,'omitnan') std(LR_Sok
,0,'omitnan') std(COV_Sok,0,'omitnan') std(SF_Sok,0,'
omitnan')  DIR_Sok;
 std(AV_Re,0,'omitnan') std(STD_Re,0,'omitnan') std(SK_Re
    ,0,'omitnan') std(KURT_Re,0,'omitnan') std(ENT_Re,0,'
    omitnan') std(ES_Re,0,'omitnan') std(LR_Re,0,'omitnan
    ') std(COV_Re,0,'omitnan') std(SF_Re,0,'omitnan')
    DIR_Re;
 std(AV_MaupA,0,'omitnan') std(STD_MaupA,0,'omitnan') std
    (SK_MaupA,0,'omitnan') std(KURT_MaupA,0,'omitnan')
    std(ENT_MaupA,0,'omitnan') std(ES_MaupA,0,'omitnan')
    std(LR_MaupA,0,'omitnan') std(COV_MaupA,0,'omitnan')
    std(SF_MaupA,0,'omitnan')  DIR_MaupA;
 std(AV_MaupB,0,'omitnan') std(STD_MaupB,0,'omitnan') std
    (SK_MaupB,0,'omitnan') std(KURT_MaupB,0,'omitnan')
    std(ENT_MaupB,0,'omitnan') std(ES_MaupB,0,'omitnan')
    std(LR_MaupB,0,'omitnan') std(COV_MaupB,0,'omitnan')
    std(SF_MaupB,0,'omitnan')  DIR_MaupB;
 std(AV_MaupC,0,'omitnan') std(STD_MaupC,0,'omitnan') std
    (SK_MaupC,0,'omitnan') std(KURT_MaupC,0,'omitnan')
    std(ENT_MaupC,0,'omitnan') std(ES_MaupC,0,'omitnan')
    std(LR_MaupC,0,'omitnan') std(COV_MaupC,0,'omitnan')
    std(SF_MaupC,0,'omitnan')  DIR_MaupC;
 std(AV_Niau,0,'omitnan') std(STD_Niau,0,'omitnan') std(
    SK_Niau,0,'omitnan') std(KURT_Niau,0,'omitnan') std(
    ENT_Niau,0,'omitnan') std(ES_Niau,0,'omitnan') std(
    LR_Niau,0,'omitnan') std(COV_Niau,0,'omitnan') std(
    SF_Niau,0,'omitnan')  DIR_Niau;
 std(AV_Ban,0,'omitnan') std(STD_Ban,0,'omitnan') std(
    SK_Ban,0,'omitnan') std(KURT_Ban,0,'omitnan') std(
    ENT_Ban,0,'omitnan') std(ES_Ban,0,'omitnan') std(
    LR_Ban,0,'omitnan') std(COV_Ban,0,'omitnan') std(
    SF_Ban,0,'omitnan')  DIR_Ban;
 std(AV_ParleA,0,'omitnan') std(STD_ParleA,0,'omitnan')
    std(SK_ParleA,0,'omitnan') std(KURT_ParleA,0,'omitnan
    ') std(ENT_ParleA,0,'omitnan') std(ES_ParleA,0,'
    omitnan') std(LR_ParleA,0,'omitnan') std(COV_ParleA
    ,0,'omitnan') std(SF_ParleA,0,'omitnan')  DIR_ParleA;
 std(AV_ParleB,0,'omitnan') std(STD_ParleB,0,'omitnan')
    std(SK_ParleB,0,'omitnan') std(KURT_ParleB,0,'omitnan
    ') std(ENT_ParleB,0,'omitnan') std(ES_ParleB,0,'
    omitnan') std(LR_ParleB,0,'omitnan') std(COV_ParleB
    ,0,'omitnan') std(SF_ParleB,0,'omitnan')  DIR_ParleB]

save StdMetrics.dat DataSTD -ascii


end



%%%%%%%%%%%%%%%%%%%%%%%%%%%%%%%%%%%%%%%%
```

```
% Extra functions
%%%%%%%%%%%%%%%%%%%%%%%%%%%%%%%%%%%%%%

function [AV,STD,SK,KURT,ENT,ES,LR,SF,COV]=Metrics(dx,L,
    varargin)

AV=[];
STD=[];
SK=[];
KURT=[];
ENT=[];
ES=[];
LR=[];
SF=[];
COV=[];

for iv=1:length(varargin)
    data=varargin{iv};
    X=data(:,1);
    Z=data(:,2);
    [Zbat]=Bathy10th(X,Z,dx,L);
    Z=Z-Zbat;
    AV=[AV mean(Z,'all')];
    STD=[STD std(Z,0,'all')];
    SK=[SK skewness(Z,1,'all')];
    KURT=[KURT kurtosis(Z,1,'all')-3];
    [p,Edges]=histcounts(Z,'Normalization','probability');
    ENT=[ENT -nansum(p.*log2(p))];
    ES=[ES EffectiveSlope1D(X,Z)];
    LR=[LR linear_roughness(X,Z)];
    SF=[SF solid_fraction(X,Z)];
    COV=[COV CoefVar(Z)];
end
end

function [Zb]=Bathy10th(X,Z,dx,L)

Span=floor(L/dx);
Xb(1)=X(1);
Zb(1)=prctile(Z(1:round(Span/4)),10);
Xb(2)=X(round(Span/4));
Zb(2)=prctile(Z(1:round(Span/2)),10);
cpt=3;
for i=round(Span/2):round(Span/4):length(X)-round(Span/2)
    ind=[i-round(Span/2)+1:i+round(Span/2)-1];
    Xb(cpt)=mean(X(ind),'omitnan');
    Zb(cpt)=prctile(Z(ind),10);
    cpt=cpt+1;
end
Xb(cpt)=max(X);
Zb(cpt)=prctile(Z(length(Z)-round(Span/2):length(Z)),10);
Zb=smooth(Zb,3);
```

```
[Xb,ind]=unique(Xb);
Zb=interp1(Xb,Zb(ind),X);
if ~isequal(size(X,1),size(Zb,1))
    Zb=reshape(Zb,1,length(Zb));
end
end

function [C]=CoefVar(Z)

for i=1:length(Z)-1
    tmp(i)=std(Z(i:i+1))/mean(Z(i:i+1));
    tmp(i)=sqrt((Z(i+1)-Z(i))^2/2)/abs((Z(i+1)+Z(i))/2);
end
C=mean(tmp,'omitnan');
end

function [ES]=EffectiveSlope1D(X,Z)

L=max(X);
dx=L/length(Z);
ES=1/L*sum(abs(gradient(Z,X))*dx);

end

function SF=solid_fraction(X,Z)

SF=(mean(Z)-min(Z))/(max(Z)-min(Z));

end
```
