# Supplementary material for: Quantifying the topographical structure of rocky and coral seabeds
Source: PLoS One. 2024 Jun 6;19(6):e0303422. doi: 10.1371/journal.pone.0303422 (PMC11156299; doi:10.1371/journal.pone.0303422)
Supplement: Attachment — Submitted filename: AnsJournalRequirements.pdf [file pone.0303422.s002.pdf]

Journal requirements

1. Please ensure that your manuscript meets PLOS ONE's style requirements, including those for file naming.

The paper has been written using LaTex template for PLoS, Version 3.6 Aug 2022.

2. Note from Emily Chenette, Editor in Chief of PLOS ONE, and Iain Hrynaszkiewicz, Director of Open Research Solutions at PLOS: Did you know that depositing data in a repository is associated with up to a 25% citation advantage (https://doi.org/10.1371/journal.pone.0230416)? If you've not already done so, consider depositing your raw data in a repository to ensure your work is read, appreciated and cited by the largest possible audience. You'll also earn an Accessible Data icon on your published paper if you deposit your data in any participating repository.

The data is avalaible online at https://doi.org/10.17882/98753

3. Please note that PLOS ONE has specific guidelines on code sharing for submissions in which author-generated code underpins the findings in the manuscript. In these cases, all author-generated code must be made available without restrictions upon publication of the work.

The data processing code has been provided in Supplementary Materials.

4. Please note that funding information should not appear in any section or other areas of your manuscript. We will only publish funding information present in the Funding Statement section of the online submission form. Please remove any funding-related text from the manuscript.

Funding information has been removed for the manuscript

5. We note that you have indicated that there are restrictions to data sharing for this study. PLOS only allows data to be available upon request if there are legal or ethical restrictions on sharing data publicly.

The data is avalaible online at https://doi.org/10.17882/98753

6. We note that Figures 1-6 in your submission contain map/satellite images which may be copyrighted. All PLOS content is published under the Creative Commons Attribution License (CC BY 4.0), which means that the manuscript, images, and Supporting Information files will be freely available online, and any third party is permitted to access, download, copy, distribute, and use these materials in any way, even commercially, with proper attribution. For these reasons, we cannot publish previously copyrighted maps or satellite images created using proprietary data, such as Google software (Google Maps, Street View, and Earth). For more information, see our copyright guidelines: http://journals.plos.org/plosone/s/licenses-and-copyright.

The orthophotographic images used for figures 1,2,5 and 6 are sourced from the IGN WMS server under the ETALAB license which is compatible with the Creative Commons' "Creative Commons Attribution" (CC-BY) . The description of this ETALAB licence can be find here: `https://www.etalab.gouv.fr/wp-content/uploads/2018/11/open-licence.pdf`

Therefore, the orthophotographic images do not fall under the category of copyrighted maps or satellite images created using proprietary data, such as Google software. As it recommended by IGN, for all publications, the source of the mapping as ©IGN 'product name' is acknowledged in the figure captions.

After reviewing your latest correspondence, we would like to provide clarification regarding the licensing terms associated with the PlanetScope imagery. Our use of PlanetScope imagery falls under the "Research License" as outlined in the Terms of Service provided by Planet Labs (`https://assets.planet.com/docs/E&R_Basic_ToS_Revised_May_2023.pdf`). As stated in the license document, the Research License explicitly

permits the use and publication of the acquired data for academic or scientific research purposes. In light of this, our understanding is that the imagery used in our manuscript falls under the category of ”free data” as defined in the Terms & Conditions provided by the European Space Agency (ESA). The use of the PlanetScope imagery aligns with the free and open data policy, meeting the criteria specified in the ESA’s Terms and Conditions. With respect to publication of Planet data, we updated the figure caption in our manuscript file to conform with the required attributions statements ’Imagery © 20xx Planet Labs’

7. Please review your reference list to ensure that it is complete and correct. If you have cited papers that have been retracted, please include the rationale for doing so in the manuscript text, or remove these references and replace them with relevant current references. Any changes to the reference list should be mentioned in the rebuttal letter that accompanies your revised manuscript. If you need to cite a retracted article, indicate the article’s retracted status in the References list and also include a citation and full reference for the retraction notice.

Reference list has been updated.
