# Supplementary material for: Quantifying the topographical structure of rocky and coral seabeds
Source: PLoS One. 2024 Jun 6;19(6):e0303422. doi: 10.1371/journal.pone.0303422 (PMC11156299; doi:10.1371/journal.pone.0303422)
Supplement: Attachment — Submitted filename: AnsRev1.pdf [file pone.0303422.s003.pdf]

Reviewer #1

We are sorry for the inconvenience caused by the incorrect figure location which has been corrected in the new version of the paper.

Best regards
